# Interesting Case of Skin Metastasis in Colorectal Cancer and Review of Literature

**DOI:** 10.1155/2018/7102845

**Published:** 2018-12-30

**Authors:** J. M. V. Amarjothi, R. Villalan, J. Jeyasudhahar, O. L. NaganathBabu

**Affiliations:** Rajiv Gandhi Government General Hospital, Department of Surgical Gastroenterology, Madras Medical College, Chennai, Tamil Nadu, India

## Abstract

Skin metastasis is a complication rarely seen after curative resection for colorectal cancer and chemotherapy. The article describes a metachronous case of skin metastasis after curative resection. This article is presented to illustrate that genetic and molecular profiling of carcinoma is a must for diagnosis of aggressive biological behavior and that skin metastasis is usually a harbinger of adverse outcome.

## 1. Introduction

Cutaneous metastases though rare may be the earliest manifestation of metastatic colorectal cancer. Such metastasis often indicates a poor prognosis, with the situation being further complicated by suboptimal treatment and aggressive biological behavior of such tumors.

**Figure 1:**
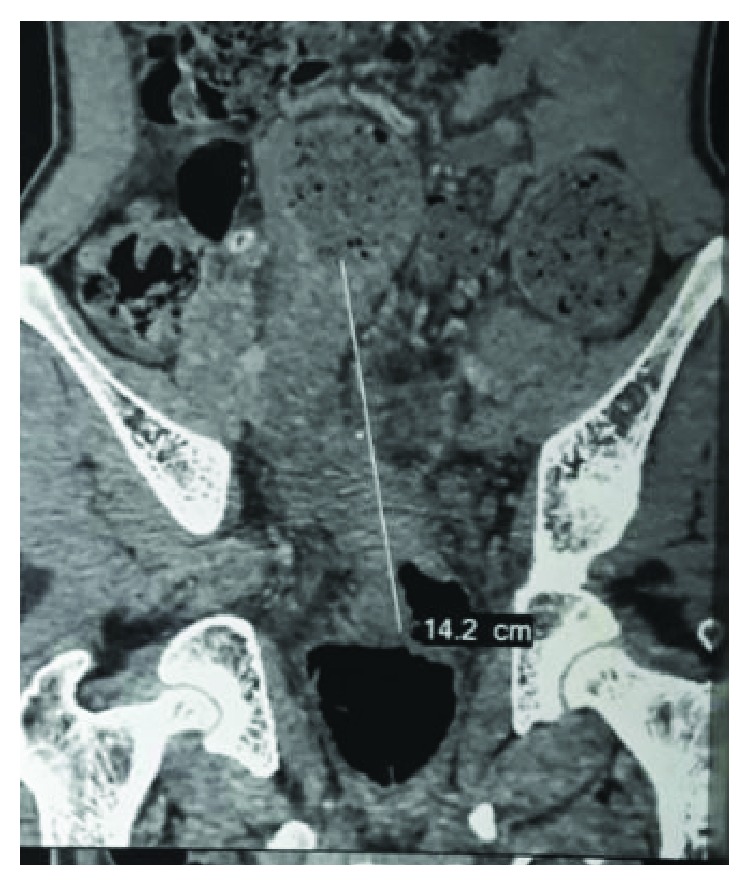
CECT showing the circumferential rectal thickening extending for 15 cm.

**Figure 2:**
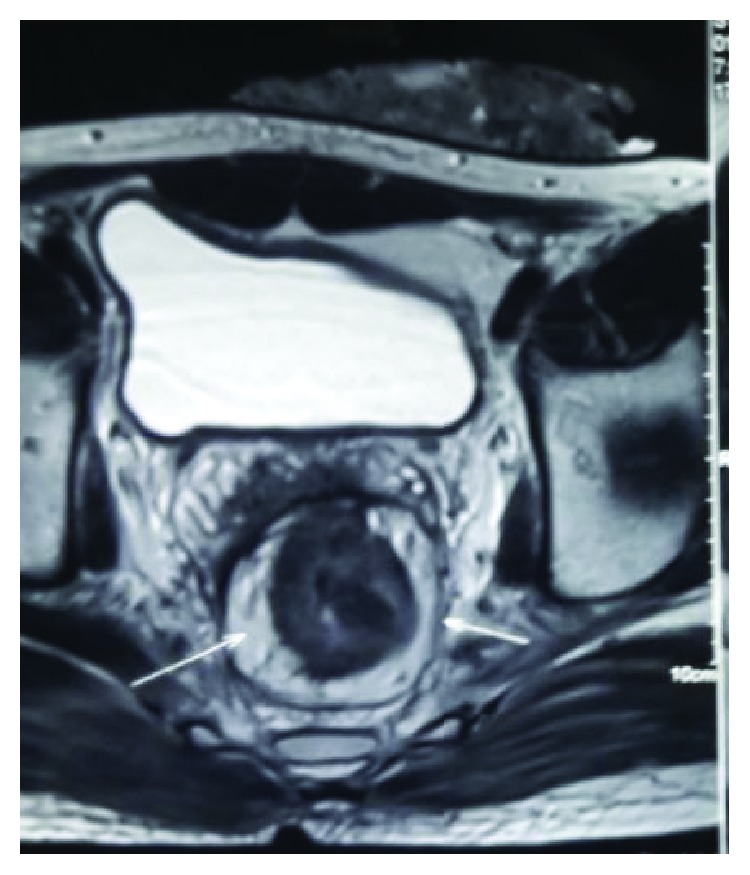
MRI pelvis of the same patient showing circumferential thickening of the rectum.

**Figure 3:**
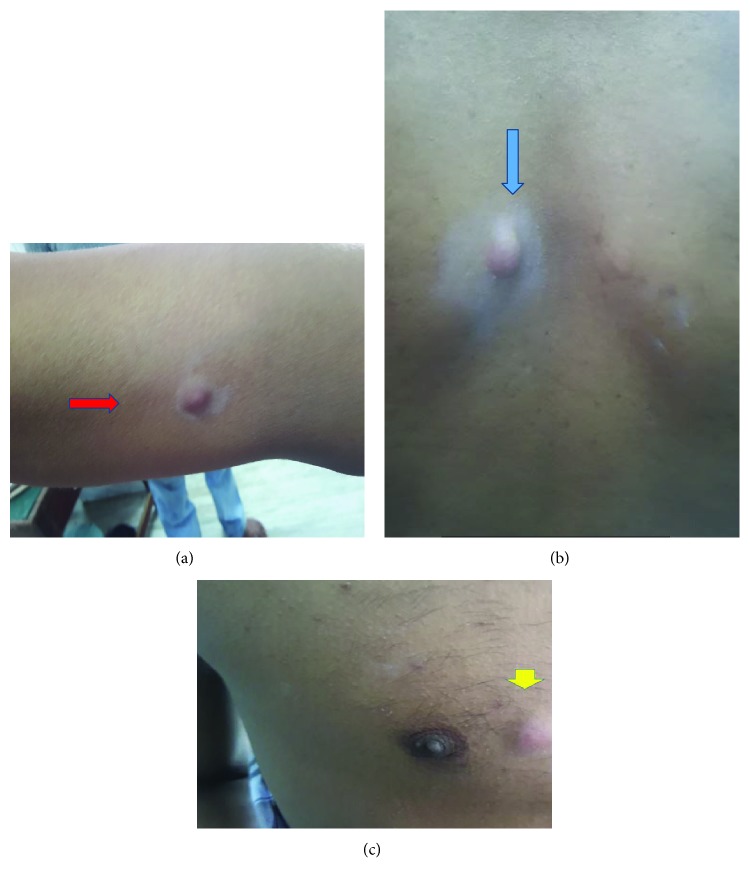
Metastatic nodules on the forearm (red arrow), chest (yellow arrow), and back (blue arrow).

A 25-year-old male patient presented with a history of bleeding per rectum, mucus discharge, and features of intestinal obstruction for 3 months. He was positive for Hepatitis B surface antigen (HBsAg); the exact etiology of which was unknown. On physical examination, rectal examination revealed semicircumferential growth involving 6 cm from anal verge 9-3 o'clock position. HPE was suggestive of poorly differentiated carcinoma. Colonoscopy was not passible due to stenosis. Carcinoembryonic antigen (CEA) levels were 1.3 ng% (<5 ng%). Contrast-enhanced computerised tomography (CECT) (Figure 1) showed irregular circumferential thickening of the wall of the rectosigmoid junction narrowing lumen, 15 cm in length from 6–19 cm with pericolonic and perirectal fat stranding. Hence, a diversion colostomy was done and the patient was subjected to long course chemoradiation with cisplatin and 5-fluorouracil and after 8-week interval, restaging was done. Per rectal, examination did not reveal palpable tumor. Imaging (Figure 2) done showed only wall thickening at the lower rectum without evidence of enlarged lymph nodes. Serum CEA was 1.7 ng% (*n* < 5 ng%); low anterior resection was done using CDH31 stapler and diversion ileostomy was done. HPE revealed complete regression of tumour in the tissue studied. The patient was put on adjuvant chemotherapy. Two months later, he developed multiple cutaneous nodules on the chest and back (Figure 3). FNAC was suggestive of adenocarcinoma. Two months later, he developed multiple peritoneal metastases and succumbed to the disease a month later.

Skin involvement is seen in about 5% of patients with colorectal cancer [1] where it appears as subcutaneous or intradermal small nodules, and it can be confused with cysts, lipomas, neurofibromas, or alopecia due to these characteristics [2, 3].

Two meta-analysis [3, 4] reported a 5–5.3% incidence of skin involvement in cancer patients. In other studies, Kauffman and Sina [5] and Lookingbill et al. [2] reported an incidence of 0.7–9% and 10%, respectively, for skin metastasis.

In an autopsy series of review of cutaneous metastasis from internal carcinoma [6, 7], the most common primary site is the breast followed by the lung. The rectum is a very rare site and the most common site of metastasis was the previous surgical scar followed by the pelvis, back, chest, upper extremities, head, and neck [5]. Most of the cutaneous metastases are well-differentiated and mucin-secreting [7]. Several mechanisms of cutaneous metastasis have been postulated including lymphatic or hematogenous spread, direct extension, or implantation during surgery [2].

Skin metastases from colorectal adenocarcinoma commonly occur metachronously within the first two years after resection of the primary tumor and are often present simultaneously with metastases to other organs like the liver [7]. The most common primary sites of cutaneous colorectal metastasis have been reported as follows: rectum (55%), sigmoid colon (17%), transverse colon (9%), rectosigmoid (7%), cecum (4%), and ascending colon (4%) [8, 9].

Skin involvement that can be seen at the time of diagnosis or during the course of treatment is a sign of advanced stage (Table 1). The prognosis is generally poor with survival of about 18 months [2] with a general range of about 1–34 months [10]. Surgical biopsy may not be logical for these patients due to poor survival and FNA cytology may be accurate for diagnosis of skin metastasis in a patient with known malignancy [11]. Wide local excision of the cutaneous metastatic lesion is the preferred treatment option in isolated lesions which is quite rare. Multiple cutaneous metastases are only palliated due to dismal prognosis [7].

## Figures and Tables

**Table 1:** Cases of rectal cancer with cutaneous metastasis.

Author, year	Age (years)	Sex	Histology	Stage	Primary cancer treatment	Interval (months)	Skin metastasis location	Skin metastasis morphology	Skin metastasis treatment	Survival (follow-up time in months)
Gottlieb and Schermer, 1970 [12]	72	F	Adenocarcinoma-sigmoid	NA	Sigmoidectomy	57	Palms	Nodules	—	NA
Gottlieb and Schermer, 1970 [12]	67	M	Adenocarcinoma-descending colon	NA	Left hemicolectomy	4	Face	Nodules/ulcers	NA	6 months
Gray and Das, 1989 [13]	79	F	Adenocarcinoma	—	Radiation	0	Leg	Nodules	None	No (18)
Reed and Stoddard, 1992 [14]	68	F	Adenocarcinoma, poorly differentiated	—	LAR	4	Perineum	Nodules	APR	—
De Friend et al., 1992 [15]	49	F	Adenocarcinoma	III	LAR	7	Perineum	Nodules	WLE	—
Kauffman and Sina, 1997 [5]	50	M	Adenocarcinoma, signet ring	IV	LAR+ACR	36	Multiple	Plaques	None	No (3)
Stavrianos et al., 2000 [16]	78	M	Adenocarcinoma-well differentiated	III	Transverse colon resection	3	Cheek oral commissure	Ulcers	RT followed by full thickness excision	11
Sukumar and Qureshi, 2001 [17]	75	M	Adenocarcinoma, poorly differentiated		APR+RT	3	Penile skin	Nodule, ulcers	NA	2
Adani et al., 2001 [18]	70	F	Adenocarcinoma	III	APR+AC	36	Leg	Nodules	CR	Yes (14)
Tsai et al., 2002 [19]	47	M	Adenocarcinoma, signet ring	III	APR+AC	11	Multiple	Nodules	C	No (4)
Melis et al., 2002 [20]	41	M	Adenocarcinoma	IV	NCR	1	Perineum	Plaques	None	—
Damin et al., 2003 [21]	44	M	Adenocarcinoma	II	LAR	6	Groin	Zosteriform	R	No (5)
Hayashi et al., 2003 [22]	50	M	Adenocarcinoma, mucinous	—	LAR	4	Perineum	Nodules	None	—
Wright et al., 2003 [23]	81	F		IV			Cholecystectomy scar			NA
Sarid et al., 2004 [24]	60	F	Adenocarcinoma, mucinous	III	NR+LAR+ACR	16	Chest, abdomen	Ulcers	WLE	No (56)
Alexandrescu et al., 2005 [25]	62	F	Adenocarcinoma	—	NA	60	Scar site	Masses	—	—
Alexandrescu et al., 2005 [25]	46	M	Adenocarcinoma	—	NA	36	Scar site	Masses	—	—
Reuter et al., 2007 [26]	69	M	Adenocarcinoma	II	APR+ACR	5	Perineum	Plaques	None	No (6)
Tan et al., 2006 [27]	70	M	Adenocarcinoma, mucinous	IIIB	LAR+AC	20	Back	Nodules	WLE, C	—
Tan et al., 2006 [27]	51	F	Adenocarcinoma	IIIB	APR	10	Perineum	Nodules	WLE, CR	9 months
Kilickap et al., 2006 [28]	29	M	Adenocarcinoma, signet ring	IIIA	LAR+APR+ACR	14	Chest wall, axilla	Nodules	WLE+C	Yes (4)
Fyrmpas et al., 2006 [29]	62	M	Adenocarcinoma-moderately differentiated	NA	Right hemicolectomy	36	Chin	Nodules	Excision biopsy	8 months
Nasti et al., 2007 [30]	76	F	Adeno carcinoma	III	Preop CRT	0	Face with parotid gland involvement	NA	NA	15
Gazoni et al., 2008 [31]	55	F	Adenocarcinoma, poorly differentiated	IV	Colostomy+CR	0	Perineum	—	CR	No (3)
Gazoni et al., 2008 [31]	66	M	Adenocarcinoma, poorly differentiated	IV	Colostomy+CR	0	Perineum	—	CR	No (4)
Gazoni et al., 2008 [31]	68	M	Adenocarcinoma, poorly differentiated	IV	Colostomy+CR	0	Thigh, axilla	—	CR	No (3)
Gazoni et al., 2008 [31]	72	M	Adenocarcinoma	IV	Colostomy+CR	0	Perineum	—	CR	No (5)
Gazoni et al., 2008 [31]	65	M	Adenocarcinoma	IV	Colostomy+CR	0	Perineum	—	CR	No (7)
Gazoni et al., 2008 [31]	78	M	Adenocarcinoma	IV	Stent+CR	0	Perineum	—	CR	No (1)
McWeeney et al., 2009 [32]	72	M	Adenocarcinoma	III	Ileostomy+NCR	6	Perineum	Nodules	WLE	—
Kurihara and Watanabe, 2009 [33]	66	M		III			Right thigh			7 months
Ayadi, 2009 [34]	63	M	Small cell carcinoma-rectum	III	CT	5	Scalp	Ulceroproliferative mass	Palliative CT	16 months
Saladzinskas et al., 2010 [35]	64	M	Adenocarcinoma, mucinous	IIA	NR+LAR	42	Face	Ulcers	WLE	Yes (7)
Ismaili et al., 2011 [36]	50	F	Adenocarcinoma, signet ring	IV	None	0	Multiple	Zosteriform	None	No (1)
Horiuchi et al., 2011 [37]	53	M	Adenocarcinoma	II		36	Scalp			6 months
Civitelli et al., 2011 [38]	73	F	Adenocarcinoma	III		Few days	Abdominal wall, chest, back			6 months
Balta et al., 2012 [39]	46	M	Adenocarcinoma, mucinous	IIIB	Colostomy	12	Perineum	Ulcers	None	—
Wang et al., 2012 [40]	63	M	Adenocarcinoma	III		6	Chest, neck, upper limb			2 weeks
Nasrolahi, 2013 [10]	33	M	Adenocarcinoma	IV	CT	3	Chest, back, neck	Plaque	CT	Few weeks
Rajan et al., 2012 [41]	36	M	Adenocarcinoma	IV		24	Lower extremities			3 months
Hamid and Hanbala, 2012 [42]	70	NA	Adenocarcinoma	II		86	Scalp, upper trunk			NA
Russo et al., 2012 [43]	72	M	Adenocarcinoma-signet cells	II	Right hemicolectomy	33	Back	Nodules	WLE	Yes
Rashid et al., 2012 [44]	65	M	Adenocarcinoma	III	Right hemicolectomy	0	Forearm	Nodules	—	17 months
de Miguel Valencia et al., 2013 [45]	55	M	Adenocarcinoma, mucinous	IIIB	NCR+APR+AC	18	Multiple	Nodules	None	No (—)
Ozgen et al., 2013 [46]	65	M	Adenocarcinoma	IIA	NCR+LAR+ACR	18	Perineum	Nodules	CR	Yes (12)
Akpak et al., 2014 [47]	47	F	Adenocarcinoma	IV	APR	36	Perineum	Ulcers	WLE+CR	—
Nesseris et al., 2013 [7]	80	M	Adenocarcinoma	III	Right hemicolectomy	12 m	Lower abdomen	Ulceroproliferative growth	2 cycles of CT	Yes
Kushwaha et al., 2013 [48]	40	M	Adenocarcinoma-signet cells	IV	CT	0	Chest, neck	Nodules	CT	4 months
Kushwaha et al., 2013 [48]	56	M	Adenocarcinoma	II	APR+CT	10	Chest, neck	Nodules	NA	8 months
Kushwaha et al., 2013 [48]	43	F	Adenocarcinoma	II	LAR+CT	8	Chest	Nodules	NA	7 months
Rogers et al., 2014 [49]	50	M	Adenocarcinoma, mucinous	IV		72	Scalp	Nodules	WLE	NA
Rogers et al., 2014 [49]	45	F	Adenocarcinoma		NCR+SX+ACR		Scalp	Nodules	WLE	Yes
Dehal et al., 2016 [50]	47	M	Adenocarcinoma, mucinous	IV	CR	1	Perineum	Nodules	R	Yes (12)
Fragulidis et al., 2015 [51]	62	M	Adenocarcinoma	IV	Endoscopic stent	4 m	Scalp	Nodules	WLE	No (2 weeks)
Varma et al., 2015 [52]	40	F	Adenocarcinoma	—	Colostomy	2 m	Pubic area, thigh	Nodules	NA	NA
Our case, 2018										

NCR: neoadjuvant chemoradiation; CR: chemoradiation; SX: surgery; CT: chemotherapy; WLE: wide local excision; NR: neoadjuvant radiation; ACR: adjuvant chemoradiation; APR: abdominoperineal resection; LAR: low anterior resection; NA: not available.

## References

[1] Balta A. Z., Sucullu I., Ozdemir Y., Dandin O. (2014). A rare clinical manifestation of rectal adenocarcinoma and synchronous scalp metastasis: a case report. *Turkish Journal of Surgery*.

[2] Lookingbill D. P., Spangler N., Helm K. F. (1993). Cutaneous metastases in patients with metastatic carcinoma: a retrospective study of 4020 patients. *Journal of the American Academy of Dermatology*.

[3] Krathen R. A., Orengo I. F., Rosen T. (2003). Cutaneous metastasis: a meta-analysis of data. *Southern Medical Journal*.

[4] Lookingbill D. P., Spangler N., Sexton F. M. (1990). Skin involvement as the presenting sign of internal carcinoma: a retrospective study of 7316 cancer patients. *Journal of the American Academy of Dermatology*.

[5] Kauffman C. L., Sina B. (1997). Metastatic inflammatory carcinoma of the rectum: tumor spread by three routes. *The American Journal of Dermatopathology*.

[6] Reingold I. M. (1966). Cutaneous metastases from internal carcinoma. *Cancer*.

[7] Nesseris I., Tsamakis C., Gregoriou S., Ditsos I., Christofidou E., Rigopoulos D. (2013). Cutaneous metastasis of colon adenocarcinoma: case report and review of the literature. *Anais Brasileiros de Dermatologia*.

[8] Hu S. C.-S., Chen G. S., Lu Y. W., Wu C. S., Lan C. C. E. (2008). Cutaneous metastases from different internal malignancies: a clinical and prognostic appraisal. *Journal of the European Academy of Dermatology and Venereology*.

[9] Brownstein M. H., Helwig E. B. (1972). Metastatic tumors of the skin. *Cancer*.

[10] Nasrolahi H., Geramizadeh B., MoaddabShoar L. (2013). Fine needle aspiration diagnosed skin metastasis in a young man with rectal cancer. *Reports of Radiotherapy and Oncology*.

[11] Geramizadeh B., Marzban S., Karamifar N., Omidifar N., Shokripour M., Mokhtareh M. (2012). Diagnosis of subcutaneous metastatic deposits by fine needle aspiration. *Journal of Cytology & Histology*.

[12] Gottlieb J. A., Schermer D. R. (1970). Cutaneous metastases from carcinoma of the colon. *Journal of the American Medical Association*.

[13] Gray M., Das S. (1989). An unusual presentation of colorectal carcinoma. *The British Journal of Clinical Practice*.

[14] Reed M. W., Stoddard C. J. (1992). Cutaneous perianal recurrence of cancer after anterior resection using the EEA stapling device. *Annals of The Royal College of Surgeons of England*.

[15] De Friend D. J., Kramer E., Prescott R., Corson J., Gallagher P. (1992). Cutaneous perianal recurrence of cancer after anterior resection using the EEA stapling device. *Annals of The Royal College of Surgeons of England*.

[16] Stavrianos S. D., McLean N. R., Kelly C. G., Fellows S. (2000). Cutaneous metastasis to the head and neck from colonic carcinoma. *European Journal of Surgical Oncology (EJSO)*.

[17] Sukumar N., Qureshi A. (2001). Adenocarcinoma of rectum metastasizing to penis. *The Medical Journal of Malaysia*.

[18] Adani G. L., Marcello D., Anania G. (2001). Subcutaneous right leg metastasis from rectal adenocarcinoma without visceral involvement. *Chirurgia Italiana*.

[19] Tsai H. L., Huang Y. S., Hsieh J. S., Huang T. J., Tsai K. B. (2002). Signet-ring cell carcinoma of the rectum with diffuse and multiple skin metastases—a case report. *The Kaohsiung Journal of Medical Sciences*.

[20] Melis M., Scintu F., Marongiu L., Mascia R., Frau G., Casula G. (2002). Inflammatory cutaneous metastasis from rectal adenocarcinoma: report of a case. *Diseases of the Colon and Rectum*.

[21] Damin D. C., Lazzaron A. R., Tarta C., Cartel A., Rosito M. A. (2003). Massive zosteriform cutaneous metastasis from rectal carcinoma. *Techniques in Coloproctology*.

[22] Hayashi H., Shimizu T., Shimizu H. (2003). Scrotal metastases originating from colorectal carcinoma. *Clinical and Experimental Dermatology*.

[23] Wright P., Jha M., Barrett P., Bain I. (2003). Colonic adenocarcinoma presenting as a cutaneous metastasis in an old operative scar. *Journal of Postgraduate Medicine*.

[24] Sarid D., Wigler N., Gutkin Z., Merimsky O., Leider-Trejo L., Ron I. G. (2004). Cutaneous and subcutaneous metastases of rectal cancer. *International Journal of Clinical Oncology*.

[25] Alexandrescu D. T., Vaillant J., Yahr L. J., Kelemen P., Wiernik P. H. (2005). Unusually large colon cancer cutaneous and subcutaneous metastases occurring in resection scars. *Dermatology Online Journal*.

[26] Reuter J., Bruckner-Tuderman L., Braun-Falco M. (2007). Epidermotropic scrotal metastasis of colorectal cancer. *International Journal of Colorectal Disease*.

[27] Tan K. Y., Ho K. S., Lai J. H. (2006). Cutaneous and subcutaneous metastases of adenocarcinoma of the colon and rectum. *Annals of the Academy of Medicine, Singapore*.

[28] Kilickap S., Aksoy S., Dinçer M., Saglam E. A., Yalçn Ş. (2006). Cutaneous metastases of signet cell carcinoma of the rectum without accompanying visceral involvement. *Southern Medical Journal*.

[29] Fyrmpas G., Barbetakis N., Efstathiou A., Konstantinidis I., Tsilikas C. (2006). Cutaneous metastasis to the face from colon adenocarcinoma. *International Seminars in Surgical Oncology*.

[30] Nasti G., Facchini G., Caraglia M. (2007). Concomitant occurrence of facial cutaneous and parotid gland metastases from rectal cancer after preoperative chemoradiotherapy. *Oncology Research and Treatment*.

[31] Gazoni L. M., Hedrick T. L., Smith P. W. (2008). Cutaneous metastases in patients with rectal cancer: a report of six cases. *The American Surgeon*.

[32] McWeeney D. M., Martin S. T., Ryan R. S., Tobbia I. N., Donnellan P. P., Barry K. M. (2009). Scrotal metastases from colorectal carcinoma: a case report. *Cases Journal*.

[33] Kurihara A., Watanabe M. (2009). Massive cutaneous metastases in the lower part of the body of a patient with rectal cancer. *Digestive Surgery*.

[34] Ayadi L., Zribi J., Mziou T. J. (2009). Scalp metastasis from small cell carcinoma of the rectum: an unusual case. *La Tunisie Medicale*.

[35] Saladzinskas Z., Tamelis A., Paskauskas S., Pranys D., Pavalkis D. (2010). Facial skin metastasis of colorectal cancer: a case report. *Cases Journal*.

[36] Ismaili Z., Dekhay S., Moussaoui A., Jahid A. (2011). Primary gastric, duodenal, and rectal signet ring cell carcinoma revealed by cutaneous metastasis. *Endoscopy*.

[37] Horiuchi A., Nozawa K., Akahane T. (2011). Skin metastasis from sigmoid colon cancer. *International Surgery*.

[38] Civitelli S., Civitelli B., Martellucci J., Tanzini G. (2011). Diffuse cutaneous metastases as the only sign of extranodal tumor spread in a patient with adenocarcinoma of the colon. *ISRN Surgery*.

[39] Balta I., Vahaboglu G., Karabulut A. A. (2012). Cutaneous metastases of rectal mucinous adenocarcinoma mimicking granuloma inguinale. *Internal Medicine*.

[40] Wang J., Shi Y.-q., Zhi-yong W. (2012). A case report of neck, chest and upper limb cutaneous metastasis from synchronous colorectal cancer. *Chinese Medical Journal*.

[41] Rajan D., Shah M., Raghavan P. (2012). Lower extremity cutaneous lesions as the initial presentation of metastatic adenocarcinoma of the colon. *Case Reports in Medicine*.

[42] Hamid G. A., Hanbala N. (2012). Adenocarcinoma of the rectum with cutaneous metastases. *Middle East Journal of Cancer*.

[43] Lo Russo G., Accarpio F., Spinelli G. P. (2012). Subcutaneous metastases from colon cancer: a case report. *Journal of Medical Case Reports*.

[44] Rashid A., Khuroo S., Nazir S., Kakroo S. M. (2012). Isolated cutaneous metastasis to forearm as a presenting feature of colon adenocarcinoma. *Journal of Pioneering Medical Sciences*.

[45] de Miguel Valencia M. J., Fraile González M., Yagüe Hernando A. (2013). Cutaneous metastases of rectal cancer. *Anales Del Sistema Sanitario De Navarra*.

[46] Ozgen A., Karakaya E., Bozdoğan N. (2013). Scrotal skin metastasis from rectum adenocarcinoma. *Rare Tumors*.

[47] Akpak Y. K., Dandin Ö., Gün I., Atay V., Haholu A. (2014). A rare case of vulvar skin metastasis of rectal cancer after surgery. *International Journal of Dermatology*.

[48] Kushwaha J. K., Sonkar A. A., Verma N., Verma K., Gupta R., Parihar A. (2013). Metastasis of rectal carcinoma in testes and skin: case series. *Indian Journal of Surgical Oncology*.

[49] Rogers J. E., Ohinata A., Dasari A., Eng C. (2014). Atypical metastatic presentations in colorectal cancer: a case series. *Clinical Colorectal Cancer*.

[50] Dehal A., Patel S., Kim S., Shapera E., Hussain F. (2016). Cutaneous metastasis of rectal cancer: a case report and literature review. *The Permanente Journal*.

[51] Fragulidis G. P., Vezakis A., Derpapas M. K., Michalaki V., Tsagkas A., Polydorou A. A. (2015). Cutaneous metastatic adenocarcinoma of the colon to the scalp. *World Journal of Oncology*.

[52] Varma K., Singh U. K., Jain M., Dhand P. L. (2015). Cutaneous metastasis in anorectal adenocarcinoma. *Indian Dermatology Online Journal*.

